# The role of human adipose-derived mesenchymal stem cell-derived exosomes in burn injury: a narrative review 

**DOI:** 10.3389/fbioe.2026.1730449

**Published:** 2026-02-02

**Authors:** Haoran Tang, Qi Wang, Jun Xue, Liang Shen, Xiaguang Duan, Biao Zhou

**Affiliations:** 1 Affiliated Third Clinical College of Inner Mongolia Medical University, Baotou, China; 2 Department of Burns and Plastic Surgery, Ulanqab Central Hospital of Inner Mongolia, Ulanqab, China

**Keywords:** burn injuries, hADSCs-Exo, mechanism, research progress, signaling pathways, wound healing

## Abstract

Burn injuries constitute a significant global health concern, presenting both health hazards and economic challenges. Recently, human adipose-derived mesenchymal stem cell exosomes (hADSCs-Exo) have emerged as a cell-free therapeutic strategy for promoting burn wound healing. These nano-sized particles function through various mechanisms, including promoting cellular migration, angiogenesis, inflammation reduction, immune response modulation, collagen remodeling, and scar prevention. They exert these effects by activating critical signaling pathways such as PI3K/Akt, IL-17RA/Smad, and mTOR. This review summarizes the biological characteristics and research relevance of hADSCs-Exo in burn injury management and discusses their translational implications.

## 1 Introduction

**FIGURE 1:**
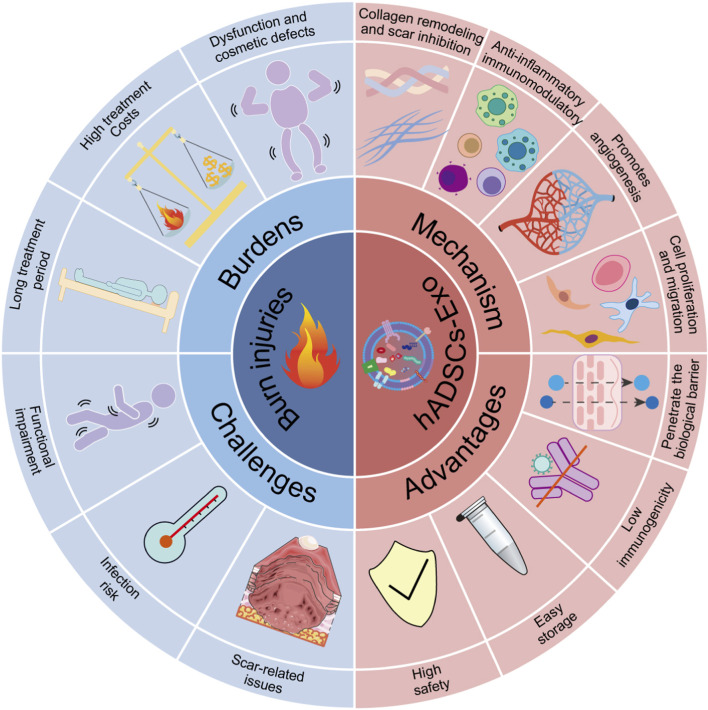
The burdens and challenges associated with burn injuries, as well as the benefits and mechanisms of hADSCs-Exo applications. The left side emphasizes the burdens and challenges of burn injuries, which include high treatment costs and prolonged rehabilitation periods, often accompanied by functional and cosmetic deficits. Challenges encompass dysfunction after wound healing, infection, and scarring issues. The right side highlights the advantages and mechanisms of hADSCs-Exo in burn injuries. The advantages include increased safety, ease of storage, good stability, low immunogenicity, reduced likelihood of rejection, and the capacity to cross biological barriers. The mechanisms involve the ability of hADSCs-Exo to enhance cellular proliferation and migration, promote neovascularization, exert anti-inflammatory and immunomodulatory effects, remodel collagen fibers, and inhibit scarring.

Burns represent a substantial global public health issue, impacting approximately 11 million individuals with severe burns and leading to over 180,000 fatalities each year (Markiewicz-Gospodarek et al., 2022). The rate of child deaths from burns is over 7 times higher in low- and middle-income countries than in high-income countries (Alemayehu et al., 2020). Burn wounds undergo a complex pathophysiological process involving multiple stages, including inflammatory response, cellular proliferation, vascular neovascularization, and tissue regeneration and remodeling (Walter et al., 2023). Severe burns significantly impair patients’ quality of life and impose substantial economic burdens and psychological stress on families. Furthermore, in clinical practice, the healing process of burn wounds encounters numerous challenges (Figure 1). With the rapid advancement of regenerative medicine, stem cell therapy offers a novel treatment strategy for burn wound repair (Table 1). Exosome therapy offers a safer, more controllable, and scalable alternative to conventional cell therapy — maintaining therapeutic potency while reducing risks tied to live cell transplantation.

**TABLE 1:** Evidence table summarizing stem cell therapy for burns.

Application performance	Specificities	Citations
Promotes angiogenesis	Stem cell transplantation significantly enhances neovascularization at burn sites, thereby accelerating oxygen and nutrient delivery to the injured area	Francis et al. (2019), Li et al. (2020), Wang M. et al. (2021)
Regulates inflammatory response and anti-inflammatory effects	Stem cells secrete multiple cytokines/growth factors to suppress excessive inflammation (e.g., by downregulating IL-1, TNF-α, etc.) and promote the establishment of a pro-reparative inflammatory environment	Henriksen et al. (2020), Wang M. et al. (2021)
Accelerates re-epithelialization and wound closure	In multiple animal models, locally applied or injected stem cells promote epithelial cell migration, wound coverage, and shorten wound closure time	Abdul Kareem et al. (2021), Ullah et al. (2021)
Promotes matrix formation/granulation tissue/collagen deposition	Stem cells promote collagen synthesis (particularly types I and III), remodeling, and ordered deposition, thereby improving the structural scaffold of wounds while facilitating the maturation and stabilization of granulation tissue	Francis et al. (2019), Ullah et al. (2021), El-Sayed et al. (2024)
Inhibits scar formation/reduces fibrosis/minimizes wound contraction	Stem cell intervention reduces collagen heterogeneity, decreases fibroblast hyperactivation, mitigates scar hyperplasia, and slows wound contraction	Lukomskyj et al. (2022), El-Sayed et al. (2024)
Facilitates regeneration of skin appendages (hair follicles, sweat glands, sebaceous glands, etc.)	Animal studies indicate that stem cell repair may extend beyond wound coverage to promote regeneration of follicle-like structures and sebaceous glands, yielding superior cosmetic and functional outcomes	Henriksen et al. (2020), Lukomskyj et al. (2022)
Mitigate burn injury progression/Prevent wound aggravation	Stem cell injection or transplantation can mitigate the “wound expansion” process. In unaffected skin areas, stem cells may prevent necrosis in adjacent regions	Ghieh et al. (2015)
Diversified local delivery methods/Carrier/Scaffold integration	Numerous studies combine stem cells with hydrogels, bio-scaffolds, nanomaterials, or ECM materials	Lukomskyj et al. (2022), El-Sayed et al. (2024), Yassaghi et al. (2024)
“Cell-free therapy” from stem cell sources: Exosomes/Conditioned media/Secretions	Exosomes secreted by stem cells, conditioned medium, or their derivatives can replace or augment stem cell therapy. This approach balances efficacy and safety, representing a promising future direction	Abdul Kareem et al. (2021), Lukomskyj et al. (2022), Das et al. (2024)
Combined with bioengineering/3D bioprinting/Nanotechnology/Guided delivery techniques	Future trends will integrate stem cells with skin bioprinting, nanocarriers, magnetically guided delivery, and smart materials to enhance targeting precision, survival rates, and functional integration	Das et al. (2024), Yassaghi et al. (2024)

Mesenchymal Stem Cells (MSCs) have become a research hotspot because of their multidirectional differentiation potential, immunomodulatory functions, and paracrine effects (Li S. et al., 2024) (Alió Del Barrio et al., 2022). hADSCs are suitable for clinical applications due to their ease of acquisition, minimal invasiveness, abundance, and strong expansion capacity. hADSCs can be obtained in large quantities from adipose tissue using minimally invasive procedures like liposuction, causing low morbidity and no ethical concerns, unlike bone marrow or embryonic sources. Exosomes are tiny messengers that carry important biologically active molecules like proteins, lipids, and nucleic acids from the mother cell. Because of this, they play a vital part in helping cells communicate, exchange substances, and share information, thereby representing a critical and highly regulated component of cellular interaction (Sun et al., 2024). hADSCs are abundant and easily harvested – adipose tissue is plentiful and can be collected with minimally invasive procedures, enabling larger-scale exosome production compared with sources like bone marrow or umbilical cord MSCs. This makes hADSC-Exo particularly attractive for clinical use (Feng et al., 2025). Compared to cell therapy, hADSCs-Exo circumvents many of the drawbacks associated with cell therapy (Zhou et al., 2023; Figure 1). In the domain of burn treatment, hADSCs-Exo demonstrates significant therapeutic potential (An et al., 2021; Song et al., 2023; Figure 1). hADSCs-Exo is poised to be a vital tool for burn wound repair, crucial for clinical translation. This review shares exciting insights into how hADSCs-Exo can help with burn injuries, paving the way for future discoveries and encouraging clinical applications.

## 2 Biological characteristics of hADSCs-Exo

### 2.1 Basic concepts and structural features of exosomes

**FIGURE 2:**
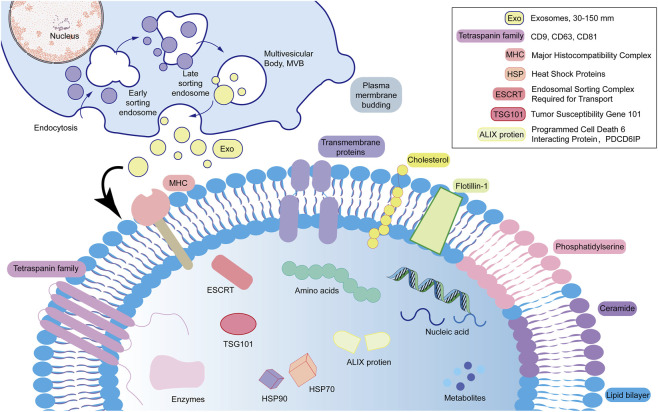
The process of exosome formation and its structural features are depicted as follows. The upper part of the figure illustrates the formation process of exosomes, initially through the invagination of the cell membrane to form early endosomes. With the entry of microRNAs (miRNAs), enzymes, and heat shock proteins into the cytoplasm, the membrane of the early endosomes is further invaginated to generate multivesicular bodies (MVBs). Ultimately, MVBs fuse with the cell membrane, releasing their internal vesicles into the extracellular environment; these vesicles are identified as exosomes. The lower section of the figure characterizes the structural composition of exosomes, which include the four transmembrane protein family (including CD9, CD63 and CD81), membrane-bound proteins, heat shock proteins (HSP, including HSP70 and HSP90), ESCRT(endosomal sorting complex required for transport) complex-associated proteins (including TSG101 and Alix), and major histocompatibility complexes (MHC, including MHC-I and MHC-II), among others. Additionally, exosomes are enriched in cholesterol, sphingomyelin, and phosphatidylserine.

Exosomes are secreted by nearly all types of cells. They are usually small, about 30–150 nm across, and often take on a cup-shaped or spherical form, made up of lipid bilayers. You can find them widely in various body fluids such as blood, lymph, urine, and cerebrospinal fluid (Mohammadinasr et al., 2024). Exosome formation is a highly regulated biological process (Sun et al., 2024; Figure 2). The exosome membrane surface is enriched with specific protein markers (Gurung et al., 2021). These proteins are not only important markers for exosome identification, but are also involved in exosome production, transport, and cellular uptake processes (Sun et al., 2017). The lipid makeup of exosome membranes is different from that of the mother cell membranes, and this unique composition helps exosomes maintain good membrane stability and are well compatible with biological systems (Zhuo et al., 2024; Figure 2).

### 2.2 Carrier characterization of hADSCs-Exo

As a natural carrier system, hADSCs-Exo gently delivers a wide range of biologically active molecules that support many important biological functions of exosomes (Table 2). This helps influence various cellular processes like gene expression, immune response, and tissue repair. Plus, they help inhibit several signaling pathways related to inflammation, cell death, and oxidative stress. The interior contents of exosomes are mainly classified into three major biomolecular groups: proteins, nucleic acids, and lipids.

**TABLE 2:** hADSCs-Exo functions, pathways, and molecular targets.

Function/Pathway	Key molecules/Targets	Citations
Promote cell proliferation/anti-apoptosis	Bcl-2, Bax, Caspase, miR-21a-3p, PI3K/AKT、mTOR signaling pathway	Zhang et al. (2018), Zhu et al. (2022)
Regulate inflammation	miR-342-3p, miR-146a,miR-21, NF-κB	Ju and Liu (2023), Wang et al. (2025)
Angiogenesis	miR-125a-3p, miR-423-5p, miR-671-3p, miR-21, VEGF/VEGFR	An et al. (2019), Wang Y. et al. (2022)
Inhibit scarring	miR-29a, miR-128-1-5p, TGF-β1/Smad、Hippo signaling pathway	Liu et al. (2022), Liang et al. (2024)
Osteogenic activity	RUNX2, ALP, SMADs, Wnt/β-catenin signaling pathway	Zhu et al. (2021), Li et al. (2023)
Remodeling of extracellular matrix	MMP-2, MMP-9	Wang Y. et al. (2022)
Modulate hypoxia response	HIF-1α,VEGF, PDGF	Wang Y. et al. (2022), Li D. et al. (2024)
Promote regeneration of skin appendages	Wnt、BMP、FGF signaling pathway	Wang Y. et al. (2022), Norouzi et al. (2024)
Nerve repair/restoration of sensory function	BDNF、NGF	Li D. et al. (2024), Norouzi et al. (2024)
Antioxidant/management of oxidative stress	Nrf2, HO-1, NQO1	Wang Y. et al. (2022)

#### 2.2.1 Protein carrier

A diverse array of functional proteins is enriched within hADSCs-Exo, encompassing membrane proteins, cytoplasmic proteins, and nuclear proteins. The membrane proteins primarily consist of four transmembrane proteins (CD63, CD81, and CD9), integrins (α4 and β1), and MHC molecules (Gurung et al., 2021). These proteins participate in the recognition and binding of exosomes to target cells. Cytoplasmic proteins encompass metabolic enzymes, signaling proteins, and cytoskeletal proteins such as β-actin, tubulin, and HSP70 (Chen et al., 2024). Nuclear proteins principally comprise transcription factors, as well as DNA and RNA-binding proteins (Hur et al., 2020). Growth factors such as vascular endothelial growth factor (VEGF), basic fibroblast growth factor (bFGF), transforming growth factor-β1 (TGF-β1) and other growth factors are enriched within hADSCs-Exo (Ding et al., 2018; Wang et al., 2018; Han et al., 2019), and matrix metalloproteinases (MMPs), tissue inhibitors of metalloproteinases (TIMPs), and other enzymes related to extracellular matrix (ECM) remodeling, and the role of these functional proteins is associated with wound healing (Ni et al., 2019).

#### 2.2.2 Nucleic acid carrier

The nucleic acids carried by hADSCs-Exo primarily consist of microRNAs (miRNAs), long non-coding RNAs (lncRNAs), messenger RNAs (mRNAs), and DNA fragments. It has been observed that hADSCs-Exo is enriched with various miRNAs associated with wound healing, including miR-21, miR-125b, miR-146a, and miR-221, miR-222 (Hade et al., 2021). miR-21, one of the most abundant miRNAs in hADSCs-Exo, has pro-angiogenic and anti-apoptotic properties (An et al., 2019; Beylerli et al., 2022). miR-125b inhibits apoptosis and promotes vascular endothelial cell function and proliferation (He et al., 2025). miR-146a exhibits anti-inflammatory properties and suppresses the NF-κB signaling pathway (Xia et al., 2025). miR-221 and miR-222 form clusters involved in the regulation of the cell cycle and angiogenesis (Celic et al., 2017). As the most important functional nucleic acids, miRNAs operate to regulate gene expression by binding to target mRNAs, thereby offering potential avenues for exosome-based treatment of burn wounds.

#### 2.2.3 Lipid component

The lipid components of hADSCs-Exo make up the membrane structure of exosomes and have important biological functions (Longo et al., 2025). Exosome membranes are rich in lipid molecules such as cholesterol, phosphatidylserine, sphingomyelin, and ceramide. These lipids not only maintain the structural integrity of exosomes but also play a role in intercellular signaling. For example, phosphatidylserine helps facilitate the uptake of exosomes by target cells (Matsumoto et al., 2017). The involvement of ceramides in the regulation of apoptosis (Pilátová et al., 2023). Lipid mediators such as prostaglandins possess anti-inflammatory properties (Wautier and Wautier, 2023). The specificity of exosomal lipid fractions is also demonstrated by their resistance to oxidative stress and their ability to protect internal contents from oxidative damage. This trait is especially important for their therapeutic potential in inflammatory settings (Chiaradia et al., 2021). In the lipidome of hADSCs-Exo, sphingolipids such as ceramides not only participate in exosome generation and targeted delivery, but also play a crucial role in antioxidant stress and anti-inflammatory responses following burns by regulating membrane microdomains, receptor clustering, and signaling pathways (Feng et al., 2025). In conclusion, the unique biological features of hADSCs-Exo make it an excellent natural therapeutic vector. Its high carrier content, superb biocompatibility, and targeting abilities provide a mechanism for burn wound regeneration. Therefore, a thorough understanding of these biological properties is vital to guide the optimization of the preparation process and improve therapeutic effectiveness.

## 3 The role of hADSCs-Exo in burn wound repair

### 3.1 Regulation of cell proliferation and migration

During the process of burn wound healing, the proliferation and migration of various cells form the core of tissue repair. hADSCs-Exo accelerates wound healing by promoting the proliferation and migration of key cell types through multiple mechanisms. (1) Fibroblasts are fundamental cells involved in wound healing and tissue repair, responsible for collagen synthesis and extracellular matrix (ECM) reconstruction. By comparing the effects of exosomes derived from different sources on human skin fibroblasts, Hoang et al. demonstrated that hADSCs-Exo enhances the migration of human skin fibroblasts more significantly (Hoang et al., 2020). hADSCs-Exo markedly facilitates fibroblast proliferation and migration through activation of the PI3K/Akt signaling pathway (Wang J. et al., 2021). (2) The proliferation and migration of epithelial cells are vital processes in wound healing and the restoration of barrier integrity. hADSCs-Exo has been shown to facilitate epithelial cell regeneration. Fu et al. discovered that hADSCs-Exo can inhibit epithelial-mesenchymal transition and fibroblast activation, thereby promoting epithelial cell regeneration (Fu et al., 2025). Exosome carrier miR-200 family members uphold the epithelial cell phenotype and inhibit epithelial-mesenchymal transition (Mirzaei et al., 2023). (3) Tissue stem cells surrounding the wound represent a critical resource for self-repair. hADSCs-Exo facilitate the activation of endogenous stem cells, including hair follicle stem cells and epidermal stem cells, thereby stimulating their proliferation and directed differentiation. Investigated the stimulatory effect of hADSCs-Exo on hair regeneration and demonstrated that hADSCs-Exo can activate hair follicle stem cells and enhance their proliferation and differentiation (Wu J. et al., 2021). hADSCs-Exo facilitates wound healing by fostering the proliferation and migration of keratinocytes (Zhang et al., 2020). The increased proliferation and migration of keratinocytes indicate that the activity of epidermal stem cells may be stimulated by hADSCs-Exo, thereby facilitating the generation and movement of keratinocytes.

### 3.2 Promotion of angiogenesis

An adequate blood supply is essential for healing burn wounds. Vascular damage remains severe after burn injuries, making vascular neovascularization crucial for delivering nutrients and removing metabolic waste. hADSCs-Exo exhibits robust pro-angiogenic properties: (1) Vascular endothelial cells serve as the primary cells involved in vascular neovascularization. It has been observed that hADSCs-Exo can enhance the proliferative capacity and lumen formation of human umbilical vein endothelial cells (HUVEC) by upregulating the expression of pro-angiogenic genes through the secretion of miR-132 and miR-146a (Heo and Kim, 2022). The study demonstrated that HUVECs treated with hADSCs-Exo exhibited the most robust lumen formation capability, with a 60%–80% enhancement in mobility relative to the control group (Hoang et al., 2020). (2) hADSCs-Exo can promote angiogenesis by activating related signaling pathways. The VEGF/VEGFR2 pathway is the primary angiogenic signal, and exosomal carrier VEGF binds to the receptor, thereby activating downstream ERK and PI3K/Akt pathways (An et al., 2021). It has been demonstrated that the co-transplantation of hADSCs-Exo with adipose tissue can activate the VEGF/AKT pathway and facilitate the vascularization of fat grafts, thereby enhancing fat survival rate (Zhu et al., 2020). The angiopoietin (Ang)-Tie pathway constitutes a principal signaling mechanism that governs angiogenesis and vascular development stability (Zhang et al., 2025). Ang-1 in exosomes plays a vital role in promoting angiogenesis and maturation (Wysoczynski et al., 2019). (3) hADSCs-Exo also promotes neovascularization under hypoxic conditions. Multiple factors contribute to microenvironmental hypoxia after burn injury. hADSCs-Exo significantly increases the survival rate of endothelial cells and reduces hypoxia-induced cell death under low oxygen conditions. It has been shown that the apoptosis rate of human dermal microvascular endothelial cells in the hypoxia/oxygen-enriched group was significantly higher, while the apoptosis rate was significantly lower after co-culturing with hypoxia-pretreated ADSCs. Additionally, co-cultivation with hypoxia-pretreated ADSCs significantly enhances cell proliferation, and the level of vascular endothelial growth factor was notably elevated (Zhao Y. et al., 2021).

### 3.3 Anti-inflammatory and immunomodulatory effects

Excessive and persistent inflammatory responses following burns are key factors that affect wound healing. hADSCs-Exo exhibits notable anti-inflammatory and immunomodulatory effects, which can regulate the severity and duration of the inflammatory response, thereby creating a microenvironment supportive of healing. hADSCs-Exo modulates the production and release of inflammatory factors through various mechanisms. (1) The exosome carrier miR-146a has anti-inflammatory properties, which can inhibit inflammatory signaling pathways and reduce the production of pro-inflammatory factors. Researchers isolated T-Exo from TNF-α-pretreated ADSCs and enriched the exosomes with miR-146a-5p, which in turn regulated the NLRP3 inflammasome pathway and thereby inhibited inflammation (Li et al., 2025). Anti-inflammatory cytokines, such as IL-10 and TGF-β, in exosomes can directly reduce inflammatory effects (Arabpour et al., 2021). (2) Macrophages are essential regulators of the inflammatory response, and their polarization status influences the direction of inflammation. hADSCs-Exo promotes macrophage polarization from M1 (pro-inflammatory) to M2 (anti-inflammatory/restorative), and studies have shown that the exocytotic carrier miR-124 can encourage M2 macrophage polarization (Yu et al., 2017; Yan et al., 2024). (3) hADSCs-Exo also regulates adaptive immune responses and maintains immune homeostasis. hADSCs-Exo inhibits T-cell overactivation and reduces cytotoxic T-cell-mediated tissue damage (Ding et al., 2025). Studies have shown that hADSCs-Exo can significantly inhibit T cell proliferation by 40%–60% (Pachler et al., 2017). It also promotes the differentiation and function of regulatory T cells (Tregs) and maintains immune tolerance (Avalos-de Leon and Thomson, 2025). Expression of exosomes can inhibit the inflammatory response by regulating Treg cells (Yan et al., 2023).

hADSCs-Exo are potent immunomodulators, capable of shifting macrophages from a pro-inflammatory M1 phenotype to an anti-inflammatory M2 phenotype (Yu et al., 2017; Yan et al., 2024). This polarization is central to their therapeutic effects in tissue repair, inflammation control, and potentially in severe systemic inflammatory conditions. hADSCs-Exo contain bioactive molecules—including proteins, cytokines, and microRNAs (miRNAs)—that collectively promote M2 macrophage polarization. This shift is characterized by decreased expression of M1 markers (e.g., iNOS, CD86) and increased expression of M2 markers (e.g., CD206, Arg1) (Heo et al., 2019; Li et al., 2022). The exosomes act through several signaling pathways, such as integrin β3/SOCS3/STAT3, S1P/SK1/S1PR1, and JAK/STAT6, to mediate these effects (Deng et al., 2019; Wang X. et al., 2022; Dong et al., 2025).

hADSCs-Exo consistently reduce pro-inflammatory cytokines (TNF-α, IL-1β, IL-6) and increase anti-inflammatory cytokines (IL-10) in both *in vitro* and *in vivo* models. This cytokine modulation underlies their anti-inflammatory and tissue-protective effects (Shen et al., 2021; Wang et al., 2025). For example, in LPS-stimulated macrophages, exosome treatment led to significant reductions in TNF-α, IL-1β, and IL-6, while boosting IL-10 production.

miR-21: Highly enriched in hADSCs-Exo, miR-21 promotes M2 polarization by downregulating pro-inflammatory pathways (e.g., NF-κB) and enhancing Akt signaling. Knockdown of exosomal miR-21 diminishes the exosome’s ability to induce M2 polarization and reduce inflammation (Rau et al., 2025). miR-146a: Targets TLR4/IRAK1/TRAF6, inhibiting NF-κB signaling and reducing TNF-α, IL-1β, and IL-6, while promoting IL-10 and M2 polarization (Rau et al., 2025). miR-125b: While not as extensively studied in the provided papers, miR-125b is implicated in modulating macrophage activation and inflammatory responses, potentially contributing to the anti-inflammatory effects of hADSCs-Exo (Dong et al., 2025).

Severe burns provoke a biphasic immune response: an immediate, massive systemic inflammatory response (SIRS) with cytokine storm, endothelial activation, coagulopathy and risk of multi-organ dysfunction; later there is a compensatory immunosuppressive phase with infection vulnerability (Willis et al., 2025). Early SIRS phase: hADSCs-Exo could dampen the cytokine storm by shifting macrophages toward M2 and lowering systemic TNF-α/IL-1β/IL-6 while increasing IL-10 — theoretically reducing organ-level inflammation, capillary leak and injury. Preclinical burn and other acute-injury models show improved local wound healing, less inflammation and better organ outcomes with MSC/ADSCs exosome treatment (Zhao et al., 2018). Later immunosuppressive phase: excessive promotion of M2/IL-10 and broad downregulation of pro-inflammatory responses could, however, deepen immune suppression and raise infection risk if given at the wrong time or dose. Thus timing and dose are crucial — dampening SIRS without tipping the balance into pathological immunosuppression is the central therapeutic challenge (Willis et al., 2025). In short: hADSCs-Exo have mechanistic and preclinical support as modulators that could reduce SIRS-related damage after severe burns, but they are not a simple “anti-inflammatory pill” — they must be applied with an eye to timing, monitored immune endpoints, and infection risk.

### 3.4 Collagen remodeling and scar prevention

**FIGURE 3:**
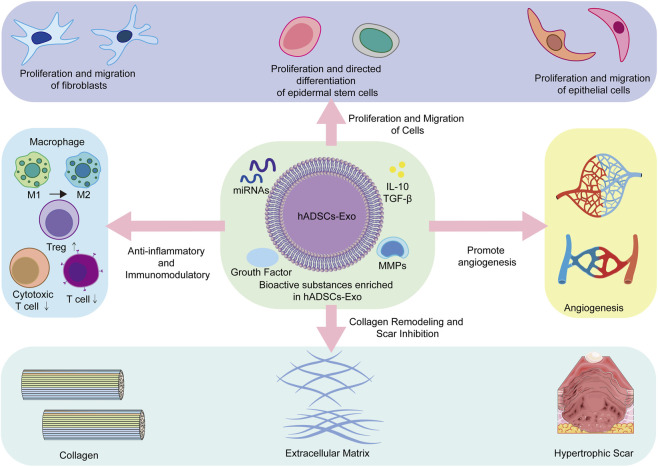
Mechanisms of hADSCs-Exo in burn injuries. The middle section shows that hADSCs-Exo carries active substances such as miRNAs, growth factors, MMPs, and the inflammatory factors IL-10, TGF-B. The upper section describes that hADSCs-Exo promotes cellular value-addition and migration, including fibroblasts, epidermal stem cells, and epithelial cells. The left half illustrates that hADSCs-Exo exerts anti-inflammatory and immunomodulatory effects, promotes polarization of M1-type macrophages to M2-type, promotes Treg, and suppresses T cells and cytotoxic T cells. The right half emphasizes that hADSCs-Exo exerts a role in promoting angiogenesis. The lower half depicts hADSCs-Exo exerting efficacy in collagen remodeling and scar inhibition.

Collagen synthesis, deposition, and remodeling are crucial aspects of wound healing, and abnormal collagen metabolism can lead to the formation of scars. Post-burn scarring is often linked to severe functional impairments and cosmetic issues, creating a significant medical burden and psychological impact. hADSCs-Exo can regulate the balance of collagen metabolism, support the restoration of normal tissues, and prevent the development of pathological scars (Li et al., 2021). (1) It has been shown that hADSCs-Exo has a dual regulation of collagen synthesis: during the early stage of wound healing, the exosome promotes collagen production for quick repair of the defect; in the later stage of healing, the exosome inhibits excessive collagen synthesis to prevent problems with scarring (Hu et al., 2016; An et al., 2021). The mechanism involves the fact that appropriate concentrations of TGF-β1 stimulate collagen production to promote tissue repair, but excess TGF-β1 leads to fibrosis. hADSCs-Exo helps maintain this balance (Wang et al., 2017). hADSCs-Exo is also enriched with regulatory genes, including miR-200, that precisely regulate collagen synthesis, preventing excessive collagen buildup during the late healing phase (Zhou et al., 2022). (2) In addition to regulating collagen synthesis, hADSCs-Exo also encourages modest collagen degradation and remodeling. Exosomes contain MMPs, with the roles of MMP-1, MMP-2, and MMP-9 in collagen breakdown being well understood. As a result, exosomes carrying these enzymes promote the breakdown of abnormal or excess collagen, which helps maintain ECM homeostasis and supports scar-free tissue repair (Ga et al., 2020; Laronha and Caldeira, 2020). The balance between MMPs and TIMPs is crucial for ECM turnover and tissue repair, and hADSCs-Exo helps maintain this balance for moderate collagen degradation (Ga et al., 2020; Zhao B. et al., 2021). (3) Pathological fibrosis is the primary cause of scar formation. hADSCs-Exo can inhibit the fibrotic process through multiple targets. Studies have shown that overexpression of miR-29a reduces collagen expression and slows proliferation (Tang et al., 2022). Thus, the members of the miR-29 family delivered by hADSCs-Exo are able to directly target and suppress the expression of collagen genes. Furthermore, the antifibrotic factor present in hADSCs-Exo counteracts the pro-fibrotic effect of TGF-β1 (Kim et al., 2024). In conclusion, hADSCs-Exo comprehensively facilitates the healing process of burn wounds through various mechanisms, including regulating cell proliferation and migration, promoting vascular neogenesis, exerting anti-inflammatory and immunomodulatory effects, and facilitating collagen remodeling (Figure 3). These mechanisms operate synergistically to sustain the dynamic equilibrium of tissue repair, thereby offering a robust theoretical foundation for the clinical utilization of hADSCs-Exo. However, current research remains focused on preclinical studies, with limited human data available (Papadopoulos et al., 2024). Therefore, more human research data is needed in the future.

## 4 Related major signaling pathways

**FIGURE 4:**
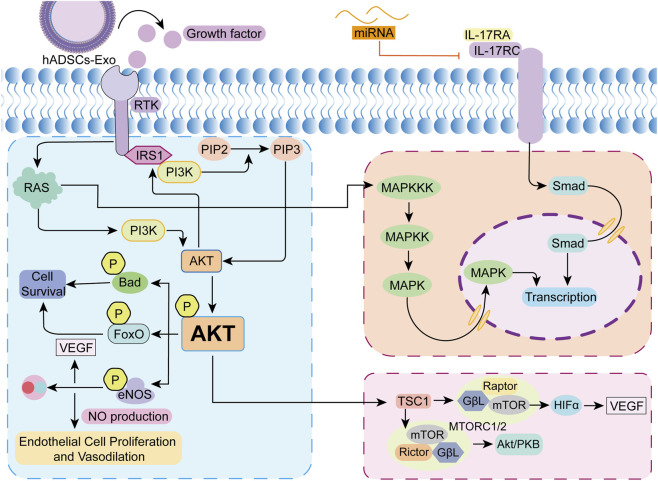
Relevant signaling pathways involved in hADSCs-Exo in Burn Injury. The left half describes the PI3K/AKT signaling pathway. When hADSCs-Exo binds to target cells, the growth factors and miRNAs it carries activate cell membrane receptors, which subsequently recruit PI3K to the cell membrane and catalyze the phosphorylation of PIP2 to generate PIP3, which serves as a second messenger that recruits Akt to the cell membrane and promotes its phosphorylation and activation, and the activated Akt regulates cell survival and proliferation through multiple downstream effector molecules, and Akt phosphorylates Bad and FoxO transcription factors to prevent apoptosis, and activates the mTORC1 complex to promote protein synthesis and cell proliferation. The upper right section describes that hADSCs-Exo contain miRNAs (e.g.,miR-192-5p). These miRNAs suppress IL-17RA protein expression by binding to the 3′ untranslated region (3′UTR) of IL-17RA mRNA, thereby inhibiting its translation or promoting its degradation. This inhibits the TGF/Smad signaling pathway, further reducing Smad expression, ultimately leading to decreased collagen synthesis and suppression of fibrosis. The lower right section highlights that AKT activated via the PI3K/AKT pathway can activate downstream MTORC1 and MTORC2, further leading to downstream VEGF activation and activation of the AKT/PKB pathway.

The role of hADSCs-Exo in burn wound repair is primarily accomplished through the activation of various essential signaling pathways, which are interconnected and collaboratively regulate biological processes, including cell proliferation, migration, differentiation, neovascularization, and tissue remodeling (Figure 4).

### 4.1 PI3K/Akt signaling pathway

The PI3K/Akt signaling pathway is one of the core regulatory pathways of hADSCs-Exo to promote burn wound healing. ADSC-derived exosomes enhance fibroblast proliferation and migration, thereby optimizing collagen deposition and accelerating wound healing through the PI3K/Akt signaling pathway (Zhang et al., 2018). ADSC-derived exosomes facilitate wound healing by promoting sebocyte regeneration through PI3K/AKT-dependent activation of sebocytes (Zhang et al., 2024). Regarding angiogenesis, Akt activates endothelial nitric oxide synthase (eNOS), which produces nitric oxide (NO), thereby promoting endothelial cell proliferation and vasodilation. Additionally, it upregulates the expression of angiogenic factors, such as vascular endothelial growth factor (VEGF), to initiate the neovascularization process response (Hu et al., 2021). It was demonstrated that pAkt expression was considerably upregulated in wound tissues treated with hADSCs-Exo, accompanied by a notable acceleration in wound healing and an increase in vascular density (Wang J. et al., 2021).

### 4.2 IL-17RA/smad signaling axis

The IL-17RA/Smad signaling axis constitutes a crucial mechanism through which hADSCs-Exo inhibit fibrosis and enhance scar quality. The regulation of this axis is primarily mediated by miR-192-5p contained within hADSCs-Exo. miR-192-5p effectively down-regulates the activation of the IL-17 signaling pathway, thereby exerting a potent anti-fibrotic effect. This subsequently results in a reduction of activity within the TGF-β/Smad signaling pathway, significantly suppressing the excessive deposition of collagens I and III, as well as the expression of α-SMA. Additionally, this mechanism diminishes the activation and proliferation of myofibroblasts, thereby limiting fibrosis driven by these cells, while also inhibiting the epithelial-mesenchymal transition (EMT) process and preserving the stability of epithelial cell phenotype (Wu Z-Y. et al., 2021). This discovery identifies a definitive molecular target for hADSCs-Exo in the intervention of pathogenic scarring.

### 4.3 mTOR signaling pathway

The mTOR signaling pathway exhibits a dual regulatory function in tissue repair mediated by hADSCs-Exo. As a principal regulator of cellular metabolism and autophagy, the pathway’s moderate activation enhances cell proliferation and protein synthesis, whereas its inhibition facilitates cellular autophagy and the removal of cellular damage. hADSCs-Exo precisely modulates metabolic reprogramming by influencing the activities of the mTORC1 and mTORC2 complexes. During the initial phase of wound healing, the controlled activation of mTORC1 encourages cellular proliferation and neovascularization. Conversely, during the stages of inflammation diminution and tissue remodeling, the downregulation of mTOR activity activates autophagy, safeguarding cells against oxidative stress damage (An et al., 2021). It was determined that the specific microRNAs contained within hADSCs-Exo (e.g., miR-21a-3p, miR-423-5p) are capable of precisely regulating the intensity of mTOR signaling. This regulation prevents cellular senescence caused by over-activation and mitigates proliferation disorders induced by excessive suppression, thus achieving optimal therapeutic outcomes (Xu et al., 2019; Fu et al., 2024).

## 5 Discussion

Exosomes and burn/skin injuries have been discussed in several previous reviews, including meta-analyses (Aghayan et al., 2025; Rong et al., 2025; Jin et al., 2025; Morabbi and Karimian, 2025; Qiao et al., 2023). However, hADSCs-Exo, as a novel acellular therapeutic approach, holds broad application prospects in the clinical treatment of burns. hADSCs-Exo represent a biologically based therapeutic modality for burn wound repair, exerting multifaceted regenerative effects that extend beyond conventional pharmacologic and cellular approaches. By integrating anti-inflammatory, pro-angiogenic, pro-regenerative, and anti-fibrotic activities, hADSCs-Exo address the complex, multistage nature of burn injury—a condition characterized by disrupted immune responses, impaired neovascularization, delayed re-epithelialization, and excessive scarring. The evidence synthesized in this review demonstrates that these effects arise from the coordinated action of exosomal cargoes, particularly miRNAs, proteins, and lipids, that converge on critical signaling pathways such as PI3K/Akt, IL-17RA/Smad, mTOR, Wnt/β-catenin, and NF-κB. These pathways collectively orchestrate the progression from early inflammation to tissue remodeling, thereby accelerating wound closure and improving functional and aesthetic outcomes. Recent research highlights a growing recognition of ongoing and completed clinical trials investigating exosome-based therapies for wound healing, particularly in chronic and diabetic wounds. A summary of ongoing clinical trials is provided in Table 3. There is clear recognition in the literature of ongoing and recent clinical trials evaluating exosome-based therapies for wound healing. Although early results are encouraging, most evidence remains derived from preclinical studies or early-phase trials. Furthermore, the majority of trials are in their initial stages or focus on diabetes/chronic wounds rather than specifically addressing burns. Therefore, adapting existing exosome clinical trial protocols to burn models necessitates key adjustments in animal model establishment, delivery methods (particularly local delivery such as exosome-hydrogels), dosing frequency, and efficacy evaluation systems. Further research is needed to determine the clinical efficacy and best practices of exosomes in burn management.

**TABLE 3:** Key ongoing and recent clinical trials on exosomes for wound healing.

Trial focus/Indication	Exosome source/Type	Phase/Status	Registry number	Citations
Diabetic chronic ulcers	MSC-exos + nutrition	Recruiting	NCT05243368	Jin et al. (2025)
Chronic cutaneous ulcers	WJ-MSC exosomes	Early Phase 1	NCT04134676	Jin et al. (2025)
Refractory cutaneous ulcers	Plasma-derived exosomes	Early Phase 1	NCT02565264	Jin et al. (2025)
Diabetic foot ulcers	Purified exosome product	Phase 2	NCT06319287	Jin et al. (2025)
Wound surface dressings	Adipose exosome + hydrogel	Not specified	NCT05475418	Jin et al. (2025)
Burns, venous ulcers, DEB	Exosome/EV therapies	Registered	NCT05078385etc.	Feng et al. (2025)
Delayed wound healing (safety)	Platelet-derived EVs	Phase 1 (completed)	ACTRN12620000944932	Johnson et al. (2023)

However, there are some limitations that need to be clarified. The major limitation of this review is its narrative nature. Systematic reviews provide higher methodological rigor. However, the current studies on hADSCs-derived exosomes in burn injury are highly heterogeneous in models, protocols, and mechanistic endpoints, making a formal systematic review or meta-analysis methodologically inappropriate at this stage. For this reason, we adopted a narrative approach to integrate mechanistic insights across diverseexperimental systems. Research on hADSCs-Exo for burn wound repair is rapidly advancing, with most evidence from animal and *in vivo* models, and limited direct human studies. Most studies involving animal models (rodents, pigs) utilize mouse or rat burn models, offering advantages such as controlled environments, reproducibility, mechanistic insights, and rapid results. Research conducted *in vivo* models overlaps with animal testing but encompasses more complex wound environments (e.g., diabetic, infected, or chronic wounds), with the benefit of closer mimicry of clinical wound complexity. Human studies are characterized by direct clinical relevance, though direct clinical trials remain scarce. Current reviews and preclinical research discuss their translational potential, but reliable human data are lacking. The discrepancies and inconsistencies in the literature primarily manifest as methodological differences and translational gaps. Methodological differences are reflected in: (1) Animal models: Studies employed different species (mice, rats, pigs), wound types (burns, diabetic, full-thickness), and comorbidities, leading to variations in healing responses and translational relevance. (2) Exosome preparation: Significant discrepancies exist in hADSCs-Exo isolation methods, dosages, and characterization, impacting reproducibility and comparability. (3) Delivery systems: Some studies employed direct injection, while others utilized hydrogels, scaffolds, or patches—methods that affect exosome retention and efficacy. Translational gaps include: (1) Species differences: Rodent skin differs structurally and in healing mechanisms from human skin, limiting direct clinical translation. (2) Lack of human data: Most evidence derives from preclinical studies; human research remains scarce, with clinical efficacy/safety yet to be established. (3) Standardization issues: No consensus exists on optimal exosome source, dose, or delivery method, leading to inconsistent results and recommendations. While hADSCs-Exo consistently shows promise for wound repair in preclinical models, methodological differences, lack of standardization, and limited human data create inconsistencies and hinder clinical translation. Future research should focus on standardized protocols, large-animal and human studies, and long-term safety to resolve these gaps.

Most available studies focus on full-thickness wound models, where hADSCs-Exo especially when delivered via hydrogels or combined with systemic administration—significantly accelerate wound closure, promote re-epithelialization, and improve overall healing quality compared to controls or stem cells alone. While some reviews suggest potential benefits across various wound types, including burns, direct head-to-head comparisons between partial- and full-thickness burns are lacking, highlighting a research gap. Therefore, hADSCs-Exo demonstrate significant potential in promoting burn wound healing, with evidence supporting their roles in angiogenesis, re-epithelialization, collagen remodeling, scar reduction, and immunomodulation. However, direct comparative studies between partial-thickness burn models and full-thickness burn models remain limited. The effects of hADSCs-Exo on angiogenesis, re-epithelialization, collagen remodeling, and scar reduction are as follows: (1) Angiogenesis: hADSCs-Exo upregulate pro-angiogenic factors (e.g., VEGF), enhance endothelial cell proliferation, and increase microvessel density in wound beds, leading to improved vascularization. (2) Re-Epithelialization: Exosomes stimulate keratinocyte proliferation and migration, accelerating re-epithelialization and restoration of the skin barrier. (3) Collagen Remodeling: Treatment increases type III collagen deposition, modulates the type III/I collagen ratio, and regulates ECM remodeling pathways, resulting in more organized collagen architecture and reduced fibrosis. (4) Scar Reduction: Exosome therapy inhibits myofibroblast activation, reduces excessive collagen deposition, and modulates signaling pathways (e.g., ERK/MAPK, Hippo), leading to decreased scar formation and improved tissue quality. (5) Immunomodulation and Inflammatory Control: Inducing M2 macrophage polarization and suppressing pro-inflammatory cytokines (e.g., TNF-α, IL-6). Modulating immune cell activity and reducing infiltration of inflammatory cells, thereby creating a regenerative microenvironment. As can be seen, direct comparative studies between partial- and full-thickness burn models are scarce. Most mechanistic and efficacy data derive from full-thickness or general wound models, underscoring the need for targeted research in partial-thickness burns and clinical translation.

The field continues to grapple with substantial heterogeneity in exosome preparations, driven by donor variability, culture conditions, isolation methods, and preconditioning strategies. This heterogeneity complicates efforts to establish potency assays, dose–response relationships, and reproducible therapeutic profiles across studies. Moreover, while numerous studies have identified key miRNAs and signaling nodes, a comprehensive systems-level understanding of exosome uptake, intracellular trafficking, and target-cell specificity in burn tissue remains lacking. The predominance of rodent models further limits translation, as these models often fail to fully replicate human burn physiology, immune architecture, and wound healing dynamics.

Safety and long-term outcomes also warrant more rigorous investigation. hADSCs-Exo are generally considered low-risk due to their non-replicating and non-immunogenic nature, yet the field lacks robust data on long-term biodistribution, clearance kinetics, repeated-dose exposure, and potential off-target effects. Equally important are the considerable manufacturing challenges that must be overcome to achieve clinical-grade production. Issues such as low yield, batch-to-batch variability, limited scalability, and absence of universally accepted characterization standards pose major barriers to regulatory approval and widespread clinical adoption. These challenges underscore the need for harmonized GMP frameworks, validated potency metrics, and standardized release criteria that align with evolving regulatory expectations for extracellular vesicle-based therapeutics.

Looking ahead, the next phase of progress will depend on coordinated innovation across basic science, bioengineering, and clinical research. Mechanistic studies should employ multi-omics profiling, advanced imaging, spatial transcriptomics, and single-cell analyses to resolve how exosome cargoes interact with diverse cellular populations in the burn microenvironment. Bioengineering solutions—including 3D bioreactors, microfluidic isolation technologies, tangential flow filtration systems, and exosome engineering approaches—will be essential for enhancing scalability, purity, and functional consistency. Integration of hADSCs-Exo into biomaterial platforms, such as hydrogels, bioactive scaffolds, and stimuli-responsive delivery systems, represents another high-potential direction for achieving sustained, localized, and stage-specific therapeutic effects. Clinically, well-designed trials that employ standardized dosing, validated biomarkers, and harmonized outcome measures are urgently needed to confirm efficacy, optimize administration protocols, and establish long-term safety. Equally important is the development of regulatory science frameworks that define product identity, potency, and therapeutic windows for exosome-based interventions in burn care.

## 6 Conclusion

In summary, the evidence synthesized in this review demonstrates that hADSCs-Exo regulate the full spectrum of burn wound repair through complex, coordinated molecular mechanisms. Their combined anti-inflammatory, pro-angiogenic, pro-regenerative, and anti-fibrotic activities position them as a central therapeutic candidate for next-generation burn management. The transition toward clinical application will require continued innovation in mechanistic elucidation, standardization, manufacturing scalability, and controlled clinical evaluation. Nonetheless, the trajectory of current research suggests that hADSCs-Exo may soon redefine regenerative strategies in severe burn care.
